# BioHastalex modified with silver nanolayers and heat treatment for antibacterial properties

**DOI:** 10.1016/j.heliyon.2024.e41467

**Published:** 2024-12-25

**Authors:** Nikola Slepičková Kasálková, Silvie Rimpelová, Cyril Vacek, Bára Frýdlová, Iva Labíková, Jan Plutnar, Kamil Severa, Václav Švorčík, Petr Slepička

**Affiliations:** a Department of Solid State Engineering, University of Chemistry and Technology Prague, 166 28 Prague, Czech Republic; b Department of Biochemistry and Microbiology, University of Chemistry and Technology Prague, 166 28 Prague, Czech Republic; c Department of Inorganic Chemistry, University of Chemistry and Technology Prague, 166 28 Prague, Czech Republic

**Keywords:** BioHastalex, Surface modification, Graphene composite, Nanostructure, Polymer stability, Surface chemistry, Morphology, Cytocompatibility

## Abstract

Here, we present surface analysis and biocompatibility evaluation of novel composite material based on graphene oxide traded as BioHastalex. The pristine material's surface morphology and surface chemistry were examined by various analytical methods. The BioHastalex with a thin silver layer was subsequently heat treated and characterized, the impact on the material surface wettability and morphology was evaluated. Significant surface roughness and morphology changes were detected at the nanometer scale after heat treatment of Ag-sputtered BioHastalex. The deposition of a thin silver nanolayer had an outstanding effect on BioHastalex's antibacterial properties while still maintaining cell viability (MRC-5, HaCaT). The heat treatment of BioHastalex-Ag led to the formation of regular nanocluster arrays while affecting the Ag concentration on the very surface. The decrease in silver concentration was connected with the length of heat treatment; cells growing on such samples exhibited good viability, and the antibacterial properties were weaker than simply sputtered BioHastalex.

## Highlights

• Novel BioHastalex polymer graphene composite has been surface enhanced.
• Heat treatment of BioHastalex-Ag led to periodic surface globular nanostructuring.
• Surface chemistry of BioHastalex was strongly affected.
• Proposed surface treatment led to antibacterial enhancement with low cytotoxicity.
• Possibility of large scale manufacturing of treated substrates was suggested.

## Graphical abstract

**Figure:**
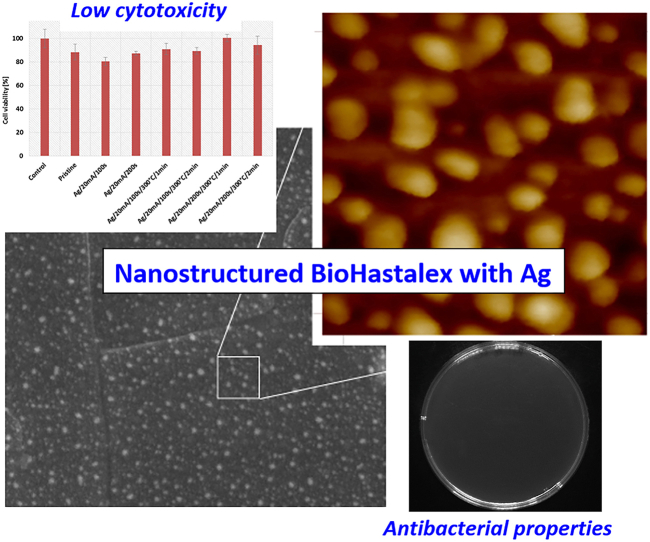

## 1 Introduction

Composite materials have been known and used for a long time. In addition to the ones commonly found in nature, such as bones and wood, they can be prepared by man, e.g., concrete belonging to one of the most used synthetic materials in the world. However, nanocomposites and graphene-based composites represent a relatively new area of research, emerging only in recent decades thanks to advances in nanotechnology [1].

Graphene-based nanocomposites, renowned for their exceptional mechanical, electrical, thermal, optical, and chemical properties [2], have a wide range of applications in fields such as biomedicine [[3], [4], [5]], electronics [6,7], energy [8,9], filtration membranes [10,11], and the automotive industry [12]. In these composite materials, the oxidized form of graphene, known as graphene oxide (GO), is often preferred. GO's superior dispersibility in composite matrices is crucial for practical applications [13]. Its functional groups help prevent the common issue of agglomeration (clustering) [14,15] seen in pristine graphene and can react with other functional groups to form covalent bonds, enhancing its incorporation into a matrix [16]. To recover some properties lost during oxidation, the oxygen-containing groups in GO can be reduced either chemically [17] or thermally [18].

A research group led by Prof. A. Seifalian has developed a novel nanocomposite material by integrating functionalized graphene oxide (FGO) nanomaterials into a backbone of poly (carbonate-urea)urethane (PCU), commercially known as Hastalex [19]. Hastalex is synthesized as a polymer solution and can be extruded into various sizes and 3D structures, including flat films and 3D shapes. This material is non-biodegradable, extremely strong, elastic, conductive, and has been proven to be nontoxic and biocompatible. Some studies have demonstrated the use of Hastalex in developing medical devices such as heart valves [19], arterial bypass grafts, cardiac patches, and urological devices including surgical membrane implants for pelvic organ prolapse [20]. Our previous article covered research on enhancing its cytocompatibility through surface activation by plasma treatment [21].

Another new nanocomposite based on functionalized graphene oxide (FGO), similar to Hastalex but biodegradable, is BioHastalex, which was engineered for broader applications in biomedical and textile fields. Thanks to its graphene content, BioHastalex is incredibly strong, lightweight, flexible, conductive, and is also biocompatible, nontoxic, biodegradable, and recyclable. In medicine, BioHastalex plays a crucial role in the NerveGraft project [22], a porous conduit designed to facilitate nerve regeneration. By integrating conductive nanofibers derived from BioHastalex and biofunctionalizing them with patient-specific peptides, growth factors, and stem cells, the technology aims to enhance regeneration. Additionally, Biohastalex is being developed as a surgical implant for pelvic organ prolapse (POP) [23], with its mechanical properties tailored to mimic pelvic tissue. This adaptation may reduce the risk of adverse reactions, such as chronic pain and inflammation, often associated with conventional polypropylene meshes, offering a safer surgical alternative. Beyond biomedical applications, BioHastalex holds promise in addressing microplastic pollution by being transformed into sustainable textiles [24]. Nanoloom, a London-based company, produces high-performance fibers and fabrics from BioHastalex for clothing. According to Nanoloom the material's biodegradability in seawater or landfill, along with its ability to be recycled back into a solution for reuse, offers significant environmental advantages [25], the Hastalex sheets are fabricated by NanoRegMed, also a London company. Given that research on Hastalex remains limited, this study aims to deepen the understanding of this groundbreaking material. In regenerative medicine and tissue engineering, biocompatibility is crucial to prevent adverse immune responses [26]. While antibacterial or bactericidal properties are desirable for preventing infections, materials must exhibit low cytotoxicity to avoid harming healthy cells [27,28].

Depending on the application, different substrate properties are required. Cytocompatible materials are essential for applications where cell growth is desired, such as in tissue engineering and regenerative medicine [29]. These materials are used in applications like wound and burn healing [30,31], bone and cartilage regeneration [32], and as scaffolds for cell growth in the creation of artificial organs including livers or kidneys [33]. Additionally, cytocompatible materials also offer exciting potential for regenerating damaged nerve tissue [34].

Conversely, in many implant applications, cell growth on biomaterials is undesirable. For example, in cardiovascular implants, excessive cell growth can lead to thrombus formation in heart valves and restenosis in stents, thus impairing blood flow [35]. In orthopedic or dental implants, undesired cell growth can cause inflammation [36], while in ocular implants, it can lead to lens opacity and visual impairment [37]. To achieve the desired effect, material properties can be tailored by surface modification techniques [38,39], which can alter characteristics [40,41] such as wettability, surface roughness, topography, chemical composition, and antimicrobial properties. All of these significantly affect the final material's biocompatibility and cell adhesion [42,43].

Cathodic sputtering is a gas-phase physical vapour deposition (PVD) technique that occurs in a vacuum chamber, thus, enabling the precise deposition of high-quality, homogeneous thin films with controlled thickness [44]. During this process, ions from the plasma bombard the cathode, causing atoms of the target material to be released. These atoms then condense on the substrate near the anode forming a thin film [45]. Thermal annealing is a key method for converting thin layers of silver (or other materials) deposited by cathodic sputtering into nanoparticle clusters [46,47]. This process involves heating the silver layer to high temperatures, which facilitates atom diffusion and the subsequent formation of nanoparticles [48]. Thermal annealing enhances the crystalline structure of the material, resulting in increased electrical conductivity and optimized optical properties [49]. The nanoparticles formed by this method are homogeneous and stable.

Alternatively, laser exposure can be used to form silver nanoparticle clusters from sputtered layers [50]. In this technique, the silver layer is exposed to laser radiation, causing local heating and the transformation of the layer into nanoparticles. Laser exposure offers precise control over the size and shape of nanoparticles [51] by fine-tuning the laser parameters, although it is a more demanding process. Silver nanoparticle clusters formed by thermal annealing and laser exposure have a wide range of applications. In biomedicine, their antibacterial properties make them ideal for coatings on medical implants and devices, where they help to minimize infection risk and promote tissue healing [52,53]. In optoelectronics, these nanoparticles enhance the performance of devices such as solar cells and sensors [54,55] thanks to their excellent optical and electrical properties [56].

In this study, we focused on a new carbon-polymer composite, BioHastalex, which has only been studied for the past three years. The surface properties resulting from various potential surface treatments are still largely unexplored. Building on our previous study on Hastalex from 2023 [21], we applied a simple surface modification technique involving the sputtering of silver onto BioHastalex. This modification led to notable changes in chemistry and moderate alterations in surface morphology, especially when combined with heat treatment. The application of a silver layer on BioHastalex significantly enhanced its antibacterial properties against both G-positive and G-negative bacteria, while still maintaining the surface viability. We are aware that Ag and its antibacterial properties are well known from the literature. The most importantly, we have conducted our research on polymer which has not been studied so far (in our previous research we have conducted experiments on Hastalex, not BioHastalex polymer). We believe strongly, that we have applied simple techniques but with outcome which brings a new interesting results for highly novel polymer composite, also with aim of cytocompatibility/viability study. This research outcomes may bring significantly new information regarding this polymer composite, its surface nanostructuring, surface enhancement and also applications in tissue engineering.

## 2 Experimental

### 2.1 Materials and modification

The studied material was a nanocomposite film (20 × 20 cm^2^, a thickness of 100 μm) with a polymer matrix and GO reinforcement called BioHastalex supplied by Goodfellow. From this film, samples of 1.5 × 1.5 cm^2^ were cut. The sputtering itself was carried out on a BAL-TEC Sputter coater SCD 050 in sputtering mode using a silver target purchased from Safina a.s. with a purity of 99.99 %. Sputtering was performed at a power of 20 mA and a distance of 55 mm from the sample to the target at a temperature of 17 °C and a pressure in the chamber of 5 Pa. The deposition time was 40–500 s.

**S**amples of pristine material and material with sputtered silver nanolayers were subjected to thermal stress in a BINDER dryer, enabling a temperature range of up to 300 °C in an open glass Petri dish at different temperatures of 100–300 °C and for different times of 1–60 min. The samples were then taken out and left to cool down to room temperature (25 °C) in the air. The thicknesses of the sputtered Ag nanolayers were determined by sputtering of glass coverslip, than the scratch was done by a special needle. The Ag thickness for layer sputtered with 20 mA and 100 s was determined to be (5.0 ± 0.3) nm, and the Ag thickness for layer sputtered with 20 mA and 200 s was determined to be (10.8 ± 1.4) nm. The errors of measurement were calculated from 3 independent scratches, on each scratch four values have been acquired, standard deviation function from MS Excel was used.

#### 2.1.1 Analytical methods

The wettability and surface energy of the samples were assessed by measuring the contact angle formed between the substrate and a liquid droplet. This was achieved using the SEE System optical device paired with Advex Instruments software. Water droplets (5 μl) and glycerol droplets (8 μl) were placed on the material surface, and images of the droplets were captured at room temperature. These images were subsequently analyzed using a three-point method to determine the contact angle values. The estimation of surface energy was also conducted using the SEE System. Two liquids, water and glycerol, were utilized for these measurements. Using the Owens-Wendt method, the contact angles were determined. These measurements were performed at room temperature. Subsequently, the surface energy values were calculated using Origin 8.0 software. The errors of measurement for contact angle were calculated from 8 independent droplets for each liquid, standard deviation function from MS Excel was used.

To investigate the surface morphology and roughness (RMS) of the samples, atomic force microscopy (AFM) was performed with a Dimension ICON microscope from BrukerCorp. Measurements were conducted in ScanAsyst mode using a silicon tip, specifically the Scan Asyst Air probe, which has a spring constant of 0.4 N m^−1^. Images were taken across areas ranging from 0.3 × 0.3 μm^2^ to 30 × 30 μm^2^.

Surface morphology was further analyzed using a scanning electron microscope (SEM) in secondary electron mode, employing the TESCAN LYRA3 GMU instrument from TESCAN (Czech Republic). SEM analysis was conducted at an accelerating voltage of 10 kV. Additionally, energy-dispersive spectroscopy (EDS) was carried out using the same equipment. Elemental composition analysis of the sample surfaces was performed using EDS with an F-MaxN analyzer and a silicon drift detector (SDD) from Oxford Instruments, UK. The acceleration voltage applied during EDS measurements was 10 kV.

X-ray photoelectron spectroscopy (XPS) was used for further analysis of the surface composition, employing a SPECS spectrometer equipped with a monochromatic Al Kα X-ray source (1486.7 eV) and a Phoibos 150 hemispherical electron analyzer. Survey spectra were acquired with a pass energy of 100 eV, while high-resolution spectra of core lines were obtained with a pass energy of 50 eV. The base chamber pressure during the measurements was maintained at 10⁻⁹ mbar or lower.

GI-XRD analysis was carried out with the use of XRDynamic 500 (Anton Paar, Austria), utilizing a Cu lamp with Kα 1.54 Å wavelength excitation (40 kV, 50 mA) and Si Pixos 2000 detector counting in 0D mode. The incident beam was set to 3°, and a parallel beam collimator was employed with a measurement radius of 360 mm. Measured diffractograms were analyzed using HighScore Plus software with the use of PDF 4+ and COD23 databases.

#### 2.1.2 Antibacterial activity

Antibacterial activity was assessed using the drop method using both Gram-negative and Gram-positive bacterial strains, specifically *E. coli* and *S. epidermidis*, respectively. The bacterial strains were initially inoculated from agar plates into liquid Luria-Bertani (LB) medium and cultured in an orbital shaker at 37 °C for 16 h. The optical densities of the bacterial cultures were then measured at 600 nm, from which serial dilutions were performed. The number of 2 × 10⁴ colony forming units (CFU) of *E. coli* and 4 × 10⁴ of S. epidermidis was inoculated into 1 mL of sterile phosphate-buffered saline (PBS, pH 7.4), into which the test samples were immersed. The samples were gently mixed and incubated at 24 °C under dynamic conditions for 6 h. Afterward, the mixtures were stirred again, and 25-μL drops from each sample (using five technical replicates) for both bacterial strains were plated onto LB agar plates in triplicates. The plates were incubated at 37 °C for 24 h, after which the CFU numbers were counted and compared to untreated controls (bacteria incubated in PBS without the tested samples). All experiments were conducted under sterile conditions.

The CFU numbers of each replicate (3 of them and each consisting of 5 CFU values from each drop) were averaged and standard deviations, expressed as error bars, were calculated using Microsoft Excel.

#### 2.1.3 Cytotoxicity evaluation

Cytotoxicity of the material 24-h leachates was performed using two adherent cell lines: human lung fibroblasts (MRC-5 pd25; Sigma-Aldrich, USA) and human keratinocytes (HaCaT; Sigma-Aldrich, USA) after 72-h treatment by a viability WST-1 assay (Sigma-Aldrich, USA). The cells were regularly passaged 2–3 times a week, i.e., when reached ca. 70 % confluency to maintain them at the exponential phase of growth. MRC-5 cells were cultivated in minimum essential medium Eagle (MEM) supplemented with 10 % (v/v) fetal bovine serum (FBS) and 1 % (v/v) of nonessential amino acid solution. The HaCaT cells were maintained in Dulbecco's modified Eagle medium (DMEM) with 10 % (v/v) FBS. Both cell lines were kept in an incubator (ThermoFisher Scientific, USA) for cell cultures at 37 °C, 5 % CO_2_ in the atmosphere and 95 % humidity. All work with cells was performed at sterile conditions using a laminar hood. To evaluate the leachate cytotoxicity, first, the number of 5000 of cells per well of 96-well plate for tissue culture (VWR) was seeded in triplicates for 24 h in 200 μL of the corresponding cell culture media, after which, the medium was changed for the sample's leachates. Cell treated only with media (no leachate added) served as a controls. After 72 h of incubation, the medium (with or without leachates) was discarded, the cells were gently washed with PBS, after which phenol red-free MEM and DMEM (for MRC-5 and HaCaT cells, respectively) was added supplemented with 4 % (v/v) of WST-1 agent (4 μL of WST-1 into 100 μL of media per well) for 2 h. After that the absorbance was measured at 450 nm (630 nm as a background) using plate spectrophotometer.

The cytotoxicity was performed in triplicates of each leachate which was prepared also in three repetitions. The responses were averaged and standard deviations, expressed as error bars, were calculated using Microsoft Excel.

## 3 Result and discussion

### 3.1 Preparation of silver nanoparticles using thermal annealing

During the thermal stress of a silver nanolayer on the surface of a polymer substrate, silver agglomeration and the nanoparticle formation can occur. To investigate this phenomenon, we prepared 1.5 × 1.5 cm^2^ Hastalex foil samples on which silver was deposited via sputtering. These samples were then heat-stressed in an open Petri dish at 300 °C for 20 min, which led to material degradation. However, for silver thicknesses corresponding to sputtering times of 40 and 100 s, a layer with a “green tint” was observed, indicating the agglomeration of silver particles and the formation of nanoparticles [57]. To optimize the preparation of this phase, additional samples with silver thicknesses corresponding to 100 s were prepared and annealed at various conditions: 100 °C/1 h, 200 °C/20, 40, 60 min, and 300 °C/1, 2, 3, 4, and 5 min. It was found that annealing at 300 °C for 5 min was optimal for silver particle agglomeration. This corresponds to the findings in Ref. [58], which reported that nanoparticles agglomerate more effectively at higher temperatures, especially with rapid annealing compared to slow heating. In our case, the phenomenona occurred with a shorter annealing time, likely due to easier polymer surface relaxation than the substrate used in the reference above. To examine the effect of the silver nanoparticles on bacteria, BioHastalex film, a biodegradable analogue of Hastalex [21], was selected as a substrate. The surface of this film was characterized before the silver modification.

### 3.2 Elemental surface analysis

**Fig. 1:**
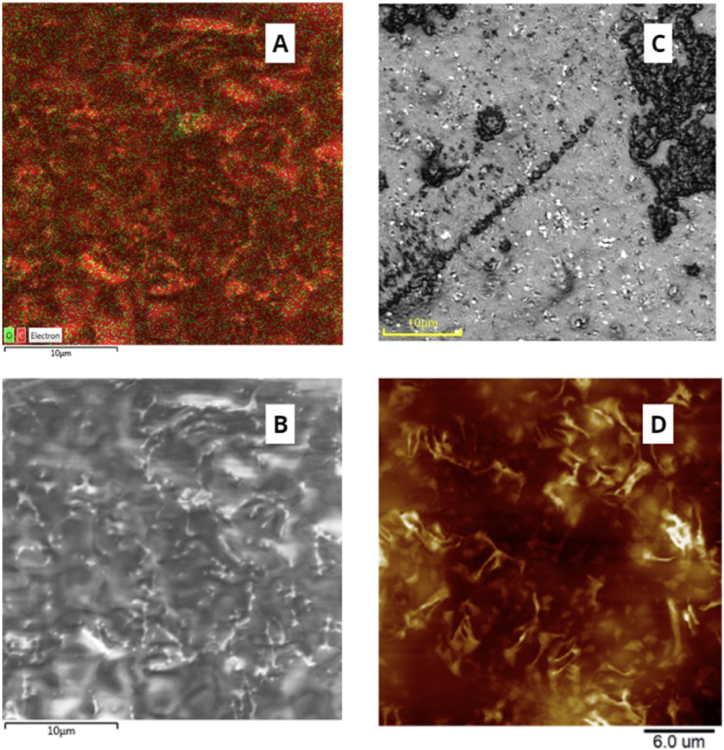
Comparison of the surface structure of pristine BioHastalex approached by different methods: A) Energy dispersive spectroscopy showing the elemental distribution on the material surface (green and red colours correspond to oxygen and carbon, respectively), B) scanning electron microscopy, C) confocal microscopy, and D) atomic force microscopy.

First, we wanted to characterize the prepared BioHastalex films (thickness of 100 μm), especially its matte side. For this purpose, we examined 1.5 × 1.5 cm^2^ BioHastalex samples by atomic force and confocal microscopy, as well as by SEM/EDS (see Fig. 1). The surface energy was calculated by measuring the water and glycerol contact angles (sessile drop method), which were 67.7 ± 2.5° and 58.4 ± 1.5°, respectively. From these data, the surface energy of the BioHastalex film was calculated using the Owens-Wendt method, the value of which was 37.2 mJ m^−2^.

### 3.3 Morphology of sputtered samples

**Fig. 2:**
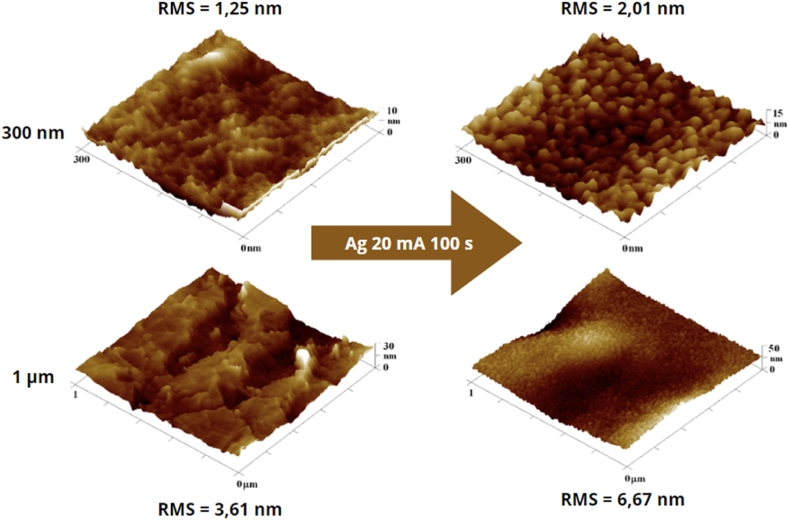
AFM scans documenting changes in surface morphology of BioHastalex samples sputtered with silver (20 mA, 100 s) recorded by 300 × 300 nm^2^ and 1 × 1 μm^2^. On the left side, there are unmodified BioHastalex films, on the right side, there are size-matched scans of the film modified with silver. The RMS values correspond to the root mean square roughness.

The effects of BioHastalex surface modification through sputtering and annealing on its morphology were examined by AFM and SEM techniques. Atomic force microscopy (AFM) scans revealed the formation of silver islands, which resulted in increased surface roughness represented by RMS value, which raised from 1.3 to 2.0 nm in the case of a 300 nm scan and from 3.6 nm to 6.7 nm in the case of a 1 μm scan. These scans are provided in Fig. 2.

**Fig. 3:**
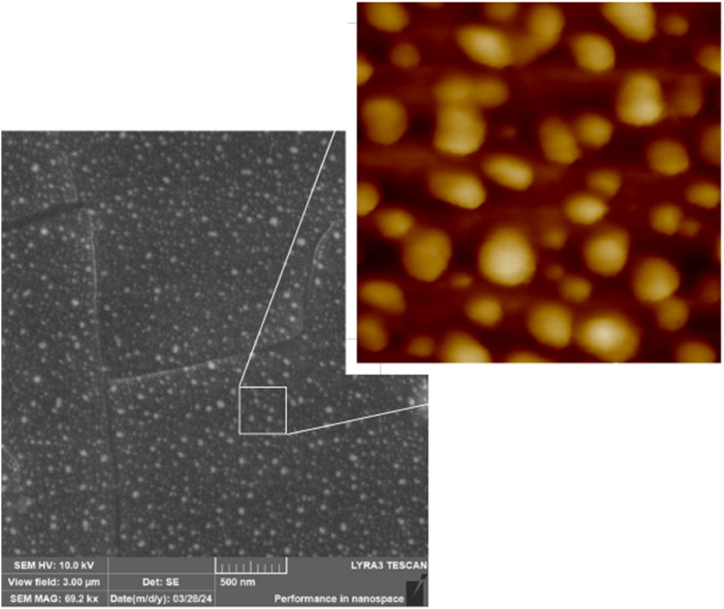
Scanning electron microscopy (SEM) image of silver nanoparticles that agglomerated on the surface of a BioHastalex film with an Ag layer deposited for 100 s at 20 mA due to thermal stress caused by 300 °C for 1 min. On the left, there is SEM image of 3 × 3 μm^2^, on the right, there is a detail captured by atomic force microscopy, area size of 300 × 300 nm^2^.

Silver modified 1.5 × 1.5 cm^2^ foil samples were further heat-stressed in a Petri dish. Due to the thinner BioHastalex film compared to the previously studied Hastalex film [21], the annealing time at 300 °C had to be reduced from 5 min to 1 or 2 min, as longer durations caused degradation. During thermal annealing (1 min) of the foil with silver layers sputtered for 100 s and 200 s, nanoparticle formation was indicated by a colour change from silvery yellow to bluish on the surface, which turned green after 2 min modification. The nanoparticles formed with a sputtering time of 100 s and 1 min of thermal stress at 300 °C are shown in Fig. 3.

**Fig. 4:**
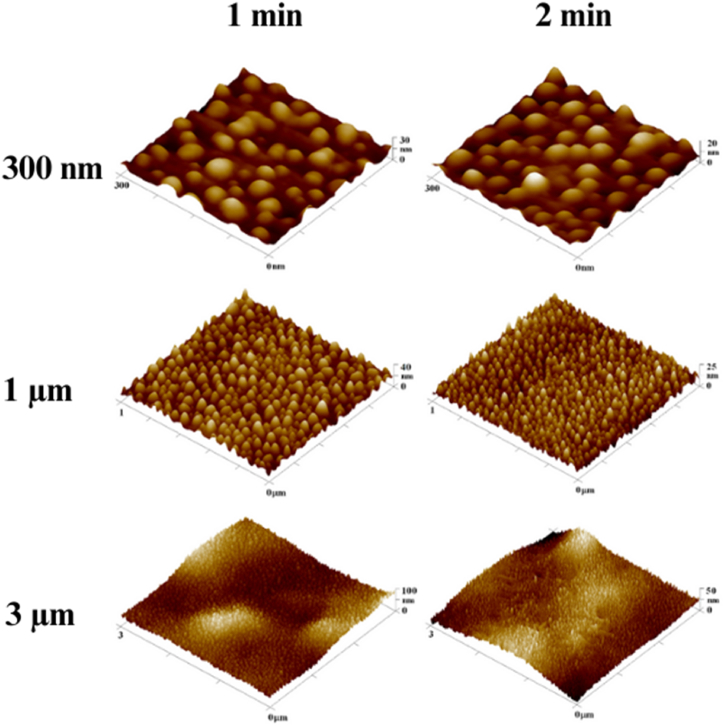
Overview of 300 × 300 nm^2^, 1 × 1 μm^2^, and 3 × 3 μm^2^ AFM scans for BioHastalex samples modified with silver for 100 s and heat stressed for 1 and 2 min at 300 °C.

**Fig. 5:**
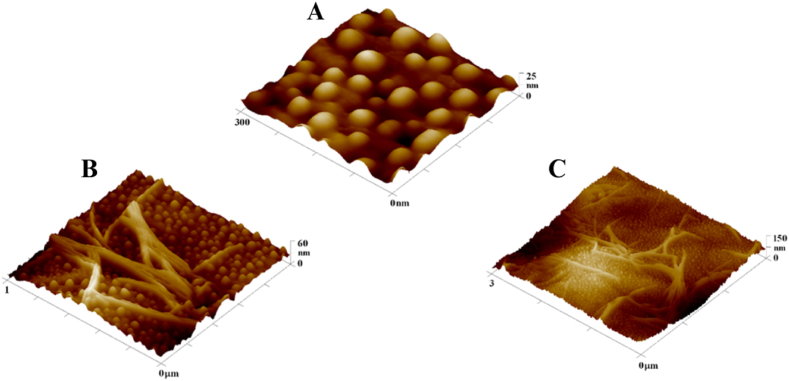
BioHastalex samples modified with silver for 100 s that were annealed at 300 °C for 3 min and document signs of degradation shown in scans from atomic force microscopy at A) 300 × 300 nm^2^, B) 1 × 1 μm^2^, and C) 3 × 3 μm^2^.

The 3D scans obtained by AFM, summarized in Fig. 4, compare the microstructures formed after depositing a silver layer for 100 s and applying heat stress for 1 and 2 min. The onset of material degradation is apparent in the 3 × 3 μm^2^ scan, indicated by the formation of lamellae on the material's surface. This likely results from the "collapse" of the Ag nanocluster-enriched surface layer and the "permeation" of material from deeper layers, which subsequently solidifies. In Fig. 5, showing AFM scans of the material after 3 min of heat stress 300 °C, the advanced stage of this modification is clearly visible, characterized by a series of lamellae. For sputtered and subsequently heated samples the nanoparticle sizes varied from 25 nm up to 40 nm.

**Fig. 6:**
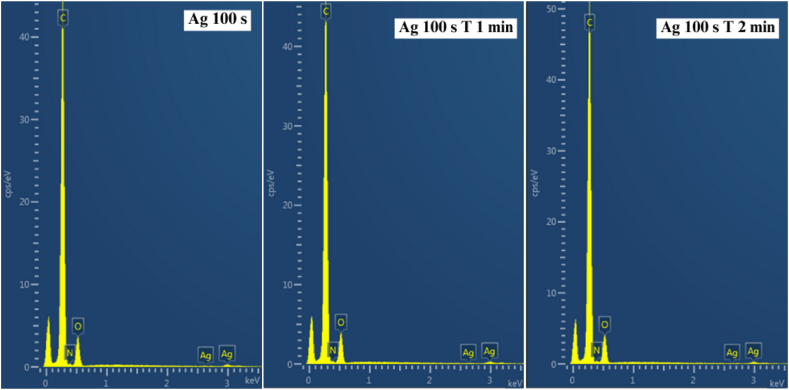
EDS spectra showing the carbon, oxygen, and silver representation on the BioHastalex surface modified by silver sputtering for 100 s and samples which were further thermally stressed at 300 °C (T) for 1 and 2 min.

As reported in previous papers, surface chemistry significantly influences the interaction between bacteria (or mammalian cells) and the biomaterial surface [[59], [60], [61], [62]]. In this case, these effects were analyzed through EDS and mass spectrometry, focusing on the concentrations of key elements. Fig. 6 presents the EDS spectra of BioHastalex with an Ag layer (100 s) and subsequently subjected to thermal stress. The corresponding mass concentrations of the monitored elements (Ag, N, C, and O) are summarized in Table 1. The data indicate that thermal stress leads to the “redistribution” of Ag on the BioHastalex surface. Additionally, with prolonged annealing, a reduction in nitrogen concentration was observed on the surface.

**Table 1:** EDS analysis of carbon, nitrogen, oxygen, and silver mass percentage (in wt. %) on the BioHastalex surface modified by silver sputtering for 100 s, the samples were further thermally stressed at 300 °C (T) for 1 and 2 min.

Sample	C (wt. %)	N (wt. %)	O (wt. %)	Ag (wt. %)
**Ag 100 s**	79.8	2.3	15.0	2.9
**Ag 100 s T 1 min**	80.9	0.3	15.9	2.9
**Ag 100 s T 2 min**	82.1	0.0	15.2	2.7

For the sake of clarity, we also show the atomic concentration of particular characterized samples. The values are introduced in Table 2. We would like to point out, that EDS analysis, even it is still considered as surface analysis, can give the information in SE (secondary mode) from up to several hundreds of nanometers, so the values from EDS analysis are not easy to compare with XPS results directly, since the absolute values of particular elements especially from very surface layers will exhibit lower absolute values compared to XPS analysis (see Table 3).

**Table 2:** EDS analysis of carbon, nitrogen, oxygen, and silver mass percentage (in at. %) on the BioHastalex surface modified by silver sputtering for 100 s, the samples were further thermally stressed at 300 °C (T) for 1 and 2 min.

Sample	C (at. %)	N (at. %)	O (at. %)	Ag (at. %)
**Ag 100 s**	85.5	2.1	11.9	0.5
**Ag 100 s T 1 min**	86.6	0.3	12.7	0.4
**Ag 100 s T 2 min**	87.5	0.0	12.1	0.4

**Fig. 7:**
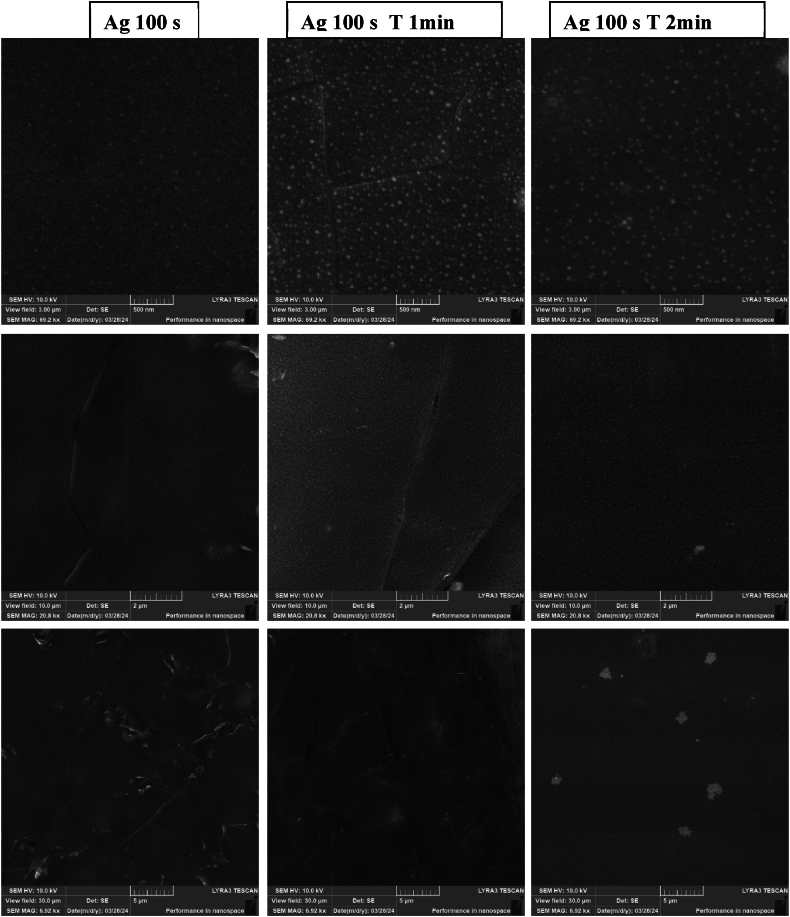
Scanning electron microscopy images of the 3, 10 and 30 μm side size of BioHastalex samples modified with silver for 100 s, and samples which were further thermally stressed at 300 °C (T) for 1 and 2 min.

Nano and microstructures formed on the BioHastalex surface, primarily through the combination of Ag deposition and subsequent thermal stress, were also examined using SEM. In the overview shown in Fig. 7, higher magnification images reveal visible clusters on the surface immediately after the silver deposition. The globular structures are induced by thermal stress applied for 1 and 2 min. The "cracks" seen in some images are likely artefacts caused by the electron beam during imaging.

### 3.4 Effect of surface modification on wettability and surface aging study

As discussed in previous studies [[59], [60], [61],[63], [64], [65]], cell adhesion to a material is influenced not only by its surface morphology but also by its chemical composition, which can change over time following modification. This phenomenon, referred to as surface aging, can be indirectly monitored through changes in surface energy, measured via contact angle. Immediately after modification – following the sputtering of a silver layer with a thickness corresponding to 100 s - the water contact angle decreased from 67.7° to 61.8°, while glycerol contact angle increased from 58.4° to 61.9°. These changes suggest an increase in surface energy from 37.2 to 40.4 mJ m^−2^. However, after thermal stress was applied for 1 min at 300 °C of the film thus modified, the surface energy decreased again to 35.0 mJ m^−2^. The effect of aging on the thus modified surface was monitored over a period of approximately 15 days post-modification (annealing). We observed that after the initial reduction of contact angles caused by annealing, the surface wettability stabilized, with no significant changes detected either in the first few hours or over the subsequent two weeks.

### 3.5 XRD analysis

**Fig. 8:**
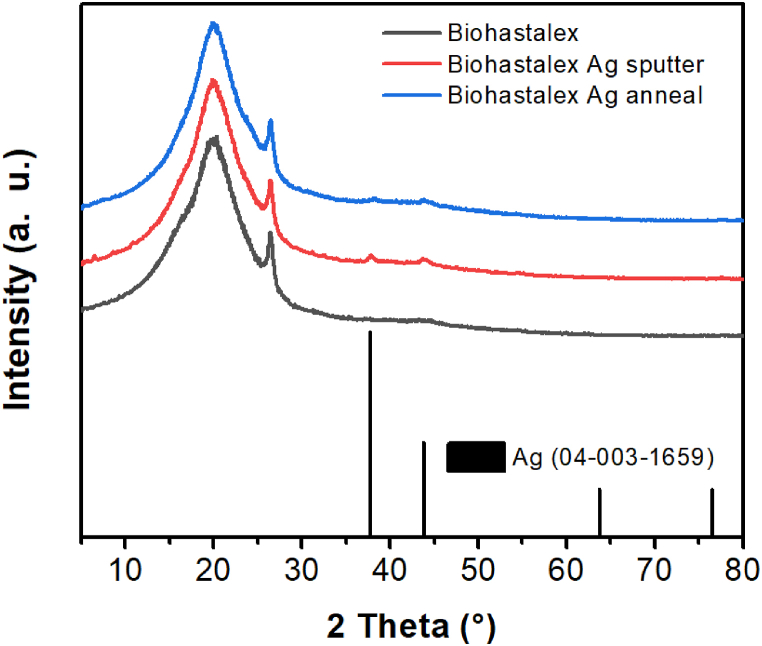
XRD diffractograms of a BioHastalex sample, a sample sputtered with silver for 100 s at 20 mA (Biohastalex Ag sputter), and the same sample subsequently heat-treated at 300 °C for 2 min (Biohastalex Ag anneal).

GI-XRD diffraction measurements (Fig. 8) of BioHastalex samples with a sputtered Ag layer, both before and after annealing, revealed the presence of cubic Ag (04-003-1659), indicated by the presence of peaks around 37.7 and 43.8°, corresponding to the (111) and (200) planes, respectively. The broadness and low intensity of those peaks are attributed to the nanocrystalline nature of the Ag layer. Consecutive annealing of the Ag layer further reduced the prominence of the Ag peaks, which may be due to Ag integration into the inner structure of the BioHastalex substrate.

### 3.6 XPS analysis

The elemental surface chemistry of a pristine BioHastalex foil and the foil sputtered with a thin silver nanolayer (Ag 100 s, 20 mA), and the same foil after heat treatment at 300 °C for 1 min and 2 min was analyzed using an XPS technique. Our primary goal was to confirm whether silver remains on the BioHastalex foil after heat treatment, and could therefore be able to contribute to antibacterial properties, and if so, what was the Ag concentration. For the simply sputtered samples, we aimed to measure the atomic concentration of silver, as previous results suggested the formation of a non-continuous Ag island field, as expected. This effect may significantly enhance antibacterial properties (via Ag island formation) but it could possibly also introduce some level of cytotoxicity.

**Fig. 9:**
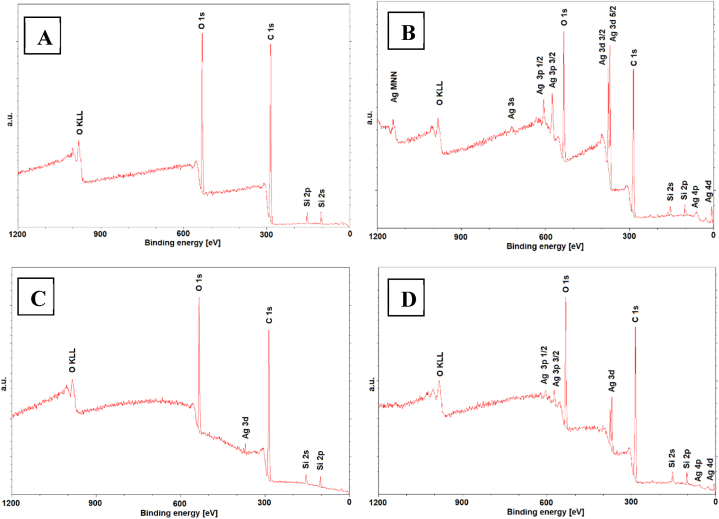
XPS spectra of a pristine BioHastalex foil (A), BioHastalex sputtered with silver for 100 s at 20 mA (B), the sputtered sample subsequently heat treated at 300 °C for 1 min. (C), and the sputtered sample subsequently heat treated at 300 °C for 2 min. (D).

Our focus was primarily on assessment of carbon, oxygen, and silver concentrations on the BioHastalex samples. The XPS spectra are given in Fig. 9, while corresponding values of elemental concentrations are introduced in (Table 3). As expected, sputtering the silver nanolayer resulted in an increase in silver atomic concentration. The very thin silver layer, in the form of nanoclusters, exhibited an atomic concentration exceeding 5 %. However, after thermal treatment, the situation changed. As previously described, the heat treatment led to the nanoisland formation but it also caused a decrease in silver atomic concentration. This suggests that silver diffused either into the thin surface layer of BioHastalex or into the newly formed surface nanoclusters. This phenomenon was confirmed by X-ray photoelectron spectroscopy, which showed a decrease in silver concentration to 0.3 at. % after 1 min of heat treatment. Interestingly, after 2 min, the concentration increased to almost 2 at. %. This increase occurred likely due to the diffusion/mass change in silver atoms/clusters within the upper BioHastalex layers. These fluctuation could be also linked to antibacterial properties of such system, which will be discussed later in the article.

**Table 3:** Atomic concentrations (at. %) of carbon, oxygen, and silver obtained by XPS on the surface of BioHastalex modified by silver sputtering for 100 s and samples further modified by thermal stress at 300 °C (T) for 1 and 2 min.

	Pristine	Ag 100 s	Ag 100 s T 1 min	Ag 100 s T 2 min
**Carbon**	73.5	67.3	71.7	70.9
**Oxygen**	21.1	19	21.6	20.1
**Silver**	–	5.2	0.3	1.9

### 3.7 Cell cytotoxicity

Fist, we aimed to assess possible cytotoxicity of BioHastalex samples and the prepared modified variants sputtered with silver for 100 and 200 s with and without subsequent heat treatment at 300 °C for 1 and 2 min. For this purpose, two model cell lines of human fibroblasts (MRC-5) and keratinocytes (HaCaT) were selected. The possible sample cytotoxicity was evaluated from their cell culture media leachates (24 h) with which the cells were treated for 72 h.

**Fig. 10:**
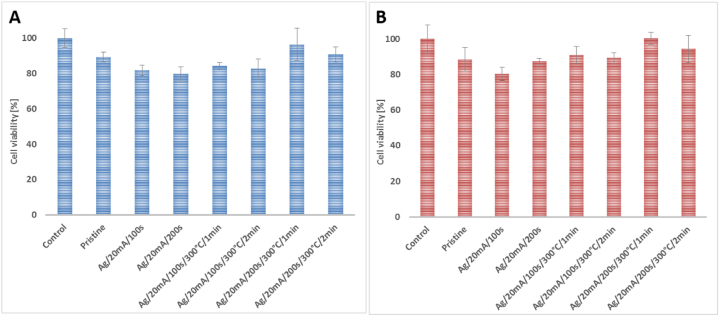
Viability of (A) human fibroblast (MRC-5) and (B) keratinocytes (HaCaT) after 72-h treatment with 24-h leachates of unmodified pristine BioHastalex film, film sputtered with silver for 100 and 200 s, and the same which was thermally stressed at 300 °C for 1 and 2 min. As a control served cells treated only with cell culture media.

From the cytotoxicity measurements depicted in Fig. 10, it is apparent, that BioHastalex leachates did not exhibit significant cytotoxicity for MRC-5 and HaCaT cell, only a moderate decrease in cell viability was detected reaching ca. a 15 % reduction, which was negligibly pronounced for Ag-sputtered samples without annealing. In contrast, viability of MRC-5 and HaCaT cells treated with leachates from Ag-sputtered BioHastalex samples which underwent further thermal annealing was comparable to that of untreated control, which makes these samples promising as biocompatible materials.

### 3.8 Antibacterial activity

**Fig. 11:**
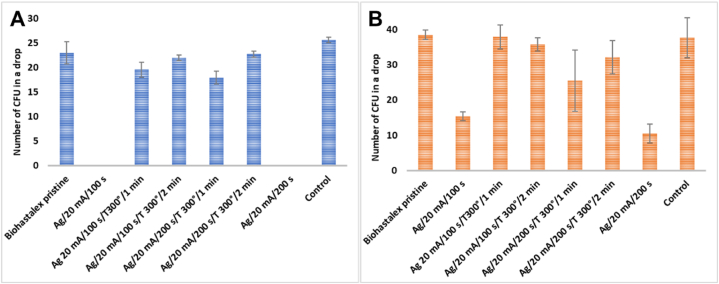
The number of colony-forming unit of (A) *E. coli* and (B) *S. epidermidis* bacteria in a drop in a 6-h contact with the tested samples: a pristine BioHastalex film, BioHastalex sputtered with silver for 100 and 200 s, and the same samples further thermally annealed at 300 °C for 1 and 2 min.

Since we found that the prepared BioHastalex materials did not induce, or only negligibly, significant cytotoxicity in MRC-5 and HaCaT cells, we advanced to evaluation of their antibacterial activity using Gram-negative bacteria *E. coli* and Gram-positive bacteria *S. epidermidis*. The results shown in Fig. 11 demonstrated a significant antibacterial effect of the sputtered silver nanolayer (without thermal annealing) which resulted in 100 % elimination of *E. coli* bacteria already after 6 h in contact with the samples. Such strong antibacterial effect of silver-modified BioHastalex on Gram-negative bacteria is consistent with our previously reported results [66]. In addition, a substantial reduction in the number of CFU of *S. epidermidis* incubated with the same samples for 6 h was detected (Fig. 11). Interestingly, however, thermal annealing of the silver layer reduced the antibacterial properties of the material. This occurred likely due to the heat-induced agglomeration of the silver islands, which exposes areas on the material surface that are not coated with silver, thus, enabling bacterial survival.

The silver is generally considered as strong antibacterial substance, however some papers indicated some limitation regarding application of Ag. Metallic silver exhibits good biocompatibility, but it is often challenging to integrate it into a nanocomposite without compromising its antibiofilm properties for optimal applications. New polymer nanocomposites (PNCs) with ultra-low filling Ag content were manufactured and tested from the point of their cytotoxicity and antibiofilm activity. It was found that PNCs with silver exhibited antibiofilm activity although they did not inhibit regular planktonic bacterial growth [67]. Silver nanoparticles synthesized via chemical reduction by using silver nitrate as the silver precursor were reported in Ref. [68], where free silver nanoparticles did not show any antibacterial activity at 125 mg/L against both *E. coli* and *S. aureus*. Limitations of recent studies dealing with the antibacterial properties of silver nanoparticles were discussed in detail in Ref. [69].

## 4 Conclusion

The main aim of this study was to modify the surface of new nanocomposite materials containing GO, BioHastalex, formed into thin films with a thickness of 100 μm, for potential applications in tissue engineering. Both unmodified BioHastalex and foils with deposited Ag layers were examined. The water and glycerol contact angles of the matte side of pristine BioHastalex were 67.7 ± 2.5° and 58.4 ± 1.5°, respectively; from them, the surface energy of 37 mJ m^−2^ was calculated. The impact of the Ag layer on surface properties, particularly roughness and morphology, was investigated in detail. It was found that depositing the Ag layer for 100 s altered the morphology, leading to cluster formation on the material surface, and an increase in surface roughness. Further, annealing studies revealed that both the temperature and duration of annealing significantly affect the substrate and the deposited layer. For instance, annealing the samples at 300 °C for just 1 or 2 min resulted in nanoparticle agglomeration, accompanied by a colour change. Prolonged annealing led to the formation of a lamellar structure. The substrate pattern may serve as master for subsequent replication into biopolymer. However, we also found that annealing did not significantly alter the surface layer. Measurements of contact angles over time, following Ag deposition and annealing demonstrated that the material modified in this way was not subject to aging, and the contact angles remained consistent over time.

We then evaluated the biological properties of the prepared samples. The antibacterial activity was assessed first, showing that the deposited silver layer (without annealing) exhibited strong antibacterial effect, eliminating nearly all *E. coli* bacteria. For *S. epidermidis*, a Gram-positive bacterium, the effect was weaker but still significant. Thermally annealed, i.e. agglomerated, layers showed reduced antibacterial potency against both bacterial strains, likely due to bacteria being trapped in the spaces between agglomerated particles. Cytotoxicity tests of the material leachates revealed that in most cases, the sputtered silver layers had little to no negative impact on human fibroblasts and keratinocytes, with only a slight decrease in cell viability observed.

## CRediT authorship contribution statement

**Nikola Slepičková Kasálková:** Writing – review & editing, Writing – original draft, Investigation. **Silvie Rimpelová:** Writing – review & editing, Investigation, Formal analysis. **Cyril Vacek:** Investigation, Data curation. **Bára Frýdlová:** Investigation, Data curation. **Iva Labíková:** Validation, Formal analysis. **Jan Plutnar:** Investigation, Data curation. **Kamil Severa:** Investigation, Data curation. **Václav Švorčík:** Methodology, Data curation. **Petr Slepička:** Writing – original draft, Supervision, Investigation, Conceptualization.

## Data availability statement

The data presented in this study are available at https://doi.org/10.5281/zenodo.14541283.

## Declaration of Competing Interest

The authors declare that they have no known competing financial interests or personal relationships that could have appeared to influence the work reported in this paper.
